# Enhancement of Thermoelectric Properties of PEDOT:PSS and Tellurium-PEDOT:PSS Hybrid Composites by Simple Chemical Treatment

**DOI:** 10.1038/srep18805

**Published:** 2016-01-05

**Authors:** Eun Jin Bae, Young Hun Kang, Kwang-Suk Jang, Song Yun Cho

**Affiliations:** 1 Division of Advanced Materials, Korea Research Institute of Chemical Technology, 141 Gajeong-ro, Yuseong-gu, Daejeon 34114, Republic of Korea

## Abstract

The thermoelectric properties of poly(3,4-ethylenedioxythiophene):poly(styrenesulfonate) (PEDOT:PSS) and tellurium-PEDOT:PSS (Te-PEDOT:PSS) hybrid composites were enhanced via simple chemical treatment. The performance of thermoelectric materials is determined by their electrical conductivity, thermal conductivity, and Seebeck coefficient. Significant enhancement of the electrical conductivity of PEDOT:PSS and Te-PEDOT:PSS hybrid composites from 787.99 and 11.01 to 4839.92 and 334.68 S cm^−1^, respectively was achieved by simple chemical treatment with H_2_SO_4_. The power factor of the developed materials could be effectively tuned over a very wide range depending on the concentration of the H_2_SO_4_ solution used in the chemical treatment. The power factors of the developed thermoelectric materials were optimized to 51.85 and 284 μW m^−1^ K^−2^, respectively, which represent an increase of four orders of magnitude relative to the corresponding parameters of the untreated thermoelectric materials. Using the Te-PEDOT:PSS hybrid composites, a flexible thermoelectric generator that could be embedded in textiles was fabricated by a printing process. This thermoelectric array generates a thermoelectric voltage of 2 mV using human body heat.

Thermoelectric devices are an attractive and environmentally friendly means to recover energy from industrial waste heat or natural heat^1^. They are efficient heat engines capable of converting a temperature difference directly into an electrical voltage via the Seebeck effect. The Seebeck coefficient is an essential indicator of the thermoelectric conversion efficiency and is the most widely measured property specific to thermoelectric materials. The performance of thermoelectric materials can be expressed as a dimensionless thermoelectric figure of merit, 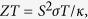 where *S* is the Seebeck coefficient, 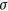 is the electrical conductivity, *κ* is the thermal conductivity, and *T* is the absolute temperature. For use in thermoelectric applications, materials should exhibit a large Seebeck coefficient as well as high electrical conductivity and low thermal conductivity. Much effort has been devoted to developing inorganic and organic thermoelectric materials with a high figure of merit, and an enhanced Seebeck coefficient and electrical conductivity in the quest to improve the thermoelectric performance of thermoelectric devices^1^.

Although inorganic materials generally exhibit high performance in thermoelectric devices, these materials are typically expensive and are characterized by brittleness, which renders their application in large areas difficult. Organic materials have unique advantages as thermoelectric materials, such as cost effectiveness, low intrinsic thermal conductivity, high flexibility, and amenability to large area applications^2^,^3^,^4^,^5^,^6^,^7^. Therefore, organic conducting polymers, which possess good electrical conductivity, have been actively researched. As mentioned above, electrical conductivity is a critical factor for assessing the performance of thermoelectric devices. Conducting polymers such as polypyrrole, polyaniline, polycarbazole, polythiophene, poly(3,4-ethylenedioxythiophene) (PEDOT), and PEDOT:poly(styrenesulfonate) (PSS) are promising candidates for thermoelectric materials^7^,^8^,^9^,^10^,^11^. Specifically, PEDOT:PSS, which is easy to handle, water-soluble, has high electrical conductivity, and offers the possibility of achieving even higher electrical conductivity, is expected to exhibit good thermoelectric performance^12^,^13^,^14^,^15^.

Prior evaluations of PEDOT:PSS as a thermoelectric material have focused on enhancing the electrical conductivity and Seebeck coefficient. Polyalcohols, ethylene glycol (EG), and dimethyl sulfoxide (DMSO) can be systematically added to PEDOT:PSS to increase the electrical conductivity^16^,^17^,^18^. Using these approaches, the electrical conductivity of PEDOT:PSS may be improved from 0.2–0.3 to 700–800 S cm^−1^, which should advantageously impact its thermoelectric performance. Recently, hydrazine solution has been used to further enhance the thermoelectric performance of PEDOT:PSS, and an electrical conductivity of 1260 S cm^−1^ was achieved for hydrazine-treated PEDOT:PSS^19^. Pipe *et al.* reported a dipping method for removing PSS from the PEDOT:PSS film using EG and DMSO, achieving a record value of *ZT* = 0.42^20^. Electrochemical reduction methods have also been used to enhance the thermoelectric performance, achieving an electrical conductivity of 1355 S cm^−1^ and a high Seebeck coefficient of 100 μV K^−1 ^21. Optimization of the *ZT* of PEDOT: *p*-toluenesulfonate by chemical reduction has also been achieved with a very high power factor of 324 μW m^−1^ K^−2^ and *ZT* = 0.25^7^,^22^. Furthermore, a high Seebeck coefficient of 163 μV K^−1^ was realized with organic-inorganic hybrid type materials, such as tellurium nanowire/PEDOT:PSS composites^23^. However, the electrical conductivity of these composites was quite poor (19.3 S cm^−1^) and resulted in unsatisfactory thermoelectric performance with a power factor of 70.9 μW m^−1^ K^−2^.

Though slight improvements in the electrical conductivity of PEDOT:PSS have been achieved with the recent approaches, further study to increase the electrical conductivity and Seebeck coefficient is still required. In particular, problems such as inaccurate control of the extent of oxidation, time-consuming procedure, and aggravation of the film surface defects in electrochemical reduction methods must be solved.

Herein, we present a convenient method for enhancing the thermoelectric properties of PEDOT:PSS by simple chemical treatment. By simply immersing thin films of the thermoelectric materials into H_2_SO_4_ solutions of various concentrations, the electrical conductivity of PEDOT:PSS can be dramatically increased^24^. To adapt this methodology for thermoelectric applications, the influence of acid treatment on the thermoelectric properties (i.e., electrical conductivity and Seebeck coefficient) of treated PEDOT:PSS is systematically investigated and analyzed. Furthermore, Te-PEDOT:PSS hybrid composites are prepared, and the effect of this chemical treatment on the thermoelectric properties of the hybrid composites is investigated. This study demonstrates that the thermoelectric performance of the Te-PEDOT:PSS composite can be greatly enhanced by tuning the electrical conductivity by acid treatment, despite the slightly decreased Seebeck coefficient. Using the treated Te-PEDOT:PSS composite having a high power factor value of 284 μW m^−1^ K^−2^, we successfully fabricated flexible thermoelectric generators by a simple printing process. This flexible thermoelectric generator is also applied to a textile-embedded-type device for application to wearable electronic devices.

## Results and Discussion

### Thermoelectric properties

**Figure 1:**
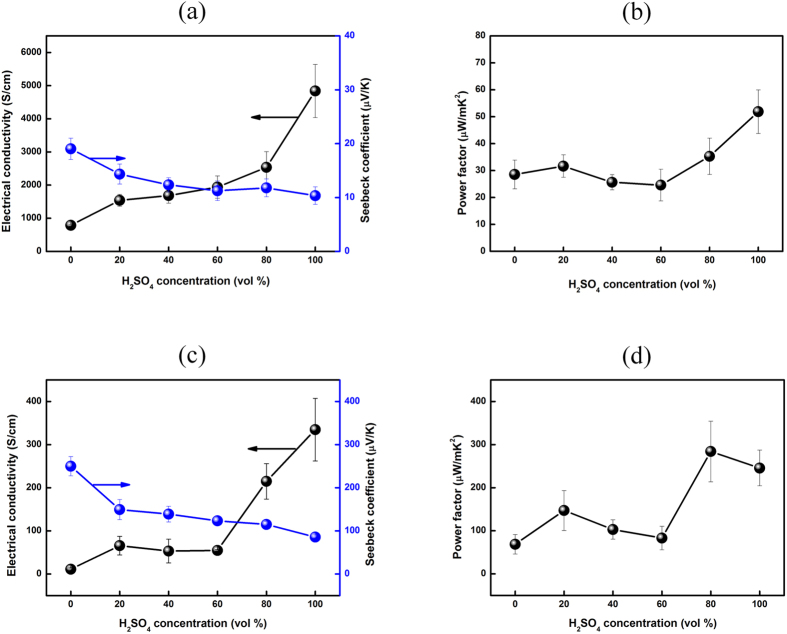
Thermoelectrical properties of the PEDOT:PSS and Te-PEDOT:PSS thin films treated with various concentrations of H_2_SO_4_. (**a**) electrical conductivity (black circles) and Seebeck coefficient (blue circles) and (**b**) power factor of PEDOT:PSS; (**c**) electrical conductivity (black circles) and Seebeck coefficient (blue circles), and (**d**) power factor of Te-PEDOT:PSS.

Simple H_2_SO_4_ chemical treatment was used to enhance the thermoelectric properties of the PEDOT:PSS thin films. The thermoelectric properties of the PEDOT:PSS thin films treated with various concentrations of H_2_SO_4_ were analyzed by measurement of the electrical conductivity, Seebeck coefficient, and power factor (*S*^*2*^*σ*) at room temperature. Figure 1(a) shows the changes in the electrical conductivity and Seebeck coefficient of the PEDOT:PSS thin films with variation of the concentration of H_2_SO_4_. The electrical conductivity increased markedly from 787.99 to 4839.92 S cm^−1^ with increasing H_2_SO_4_ concentration, whereas the Seebeck coefficient decreased from 19.4 to 10.35 μV K^−1^. Figure 1(b) shows the power factor of PEDOT:PSS after treatment with various concentrations of H_2_SO_4_. The average power factor was 33.81 μW m^−1^ K^−2^ and the optimized power factor of the PEDOT:PSS thin films was 51.85 μW m^−1^ K^−2^, achieved with a H_2_SO_4_ concentration of 100 vol%. The power factor of H_2_SO_4_-treated PEDOT:PSS is much higher than that of non-treated PEDOT:PSS due to the notable enhancement of the electrical conductivity of H_2_SO_4_-treated PEDOT:PSS.

As previously reported, Te-PEDOT:PSS hybrid composites have a high Seebeck coefficient due to the nature of the Te nanorods but their electrical conductivity is unacceptably low^23^. To overcome this poor electrical conductivity, we synthesized Te nanorods encapsulated with PEDOT:PSS^23^ and treated them with various concentrations of H_2_SO_4_. The formation of the nanocrystalline structure of Te-PEDOT:PSS hybrids was confirmed by transmission electron microscopy (TEM) as shown in Fig. S1. The Te-PEDOT:PSS hybrids consist of Te nanorods (diameter: 20–30 nm; length: ca. 800 nm) coated with a thin PEDOT:PSS layer. Smooth and uniform films of the Te-PEDOT:PSS hybrid composite were easily formed by drop casting.

The effect of H_2_SO_4_ treatment on the thermoelectric properties of the Te-PEDOT:PSS hybrid films was evaluated by immersion of the films in H_2_SO_4_ of various concentrations. The electrical conductivity, Seebeck coefficient, and power factor of the treated thin films as a function of the H_2_SO_4_ concentration are shown in Fig. 1(c,d). Although the Seebeck coefficient of the Te-PEDOT:PSS hybrid composite films decreased with increasing H_2_SO_4_ concentration, the electrical conductivity was significantly enhanced from 11.01 to 334.68 S cm^−1^. The optimized power factor of the H_2_SO_4_-treated Te-PEDOT:PSS hybrid films was determined to be 284 μW m^−1^ K^−2^ at a H_2_SO_4_ concentration of 80 vol%.

The respective estimated *ZT* values for H_2_SO_4_-treated PEDOT:PSS and Te-PEDOT:PSS based on the thermal conductivity of PEDOT:PSS and Te-PEDOT:PSS documented in the literature were 0.08 and 0.39^6^,^23^,^24^,^25^,^26^,^27^. The electrical conductivity, Seebeck coefficient, optimized power factor, and estimated *ZT* value of H_2_SO_4_-treated PEDOT:PSS and Te-PEDOT:PSS are summarized in Table 1.

### Electrical conductivity

**Figure 2:**
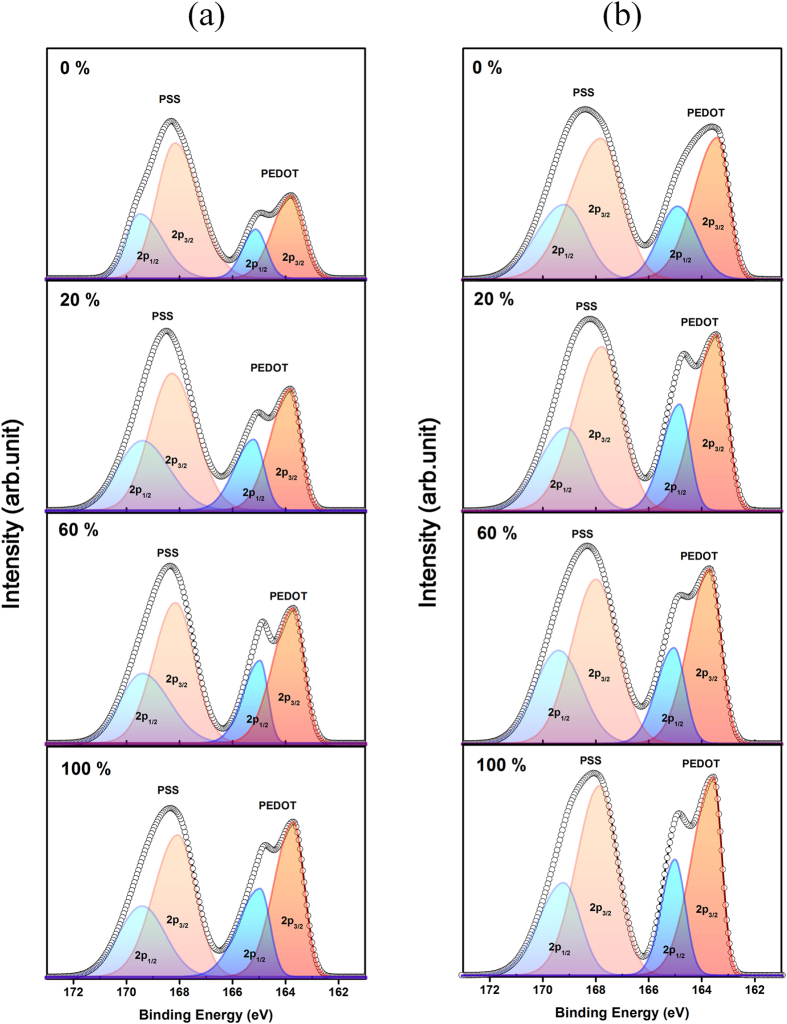
XPS (S2p) spectra of (**a**) PEDOT:PSS and (**b**) Te-PEDOT:PSS hybrid composite films treated with various concentrations of H_2_SO_4_.

The enhanced electrical conductivity of H_2_SO_4_-treated PEDOT:PSS and Te-PEDOT:PSS is believed to arise from structural rearrangement of PEDOT:PSS due to the removal of PSS, which induces the formation of a more crystalline structure^27^,^28^. The improved crystallinity of PEDOT:PSS leads to an increase in the electrical conductivity. To confirm the ability of H_2_SO_4_ treatment to induce conformational and compositional changes of the PEDOT:PSS and Te-PEDOT:PSS films, the surface chemical compositions of the films were analyzed by X-ray photoelectron spectroscopy (XPS). Figure 2 demonstrates that the PEDOT:PSS and Te-PEDOT:PSS films treated with H_2_SO_4_ of various concentrations exhibit S2p peaks characteristic of two distinct types of sulfur atoms because the sulfur atoms of the thiophene unit in PEDOT and of the sulfonate group in PSS have different binding energies. The broad peak at higher binding energy can be attributed to overlap of the S2p_3/2_ and S2p_1/2_ peaks (168.1 eV and 169.4 eV) of the S atoms in PSS, while the doublet S2p_3/2_ and S2p_1/2_ peaks (163.7 eV and 165.1 eV) at lower binding energy arise from the S atoms in PEDOT^29^,^30^,^31^,^32^. The higher S2p binding energy of PSS is due to the electronegative oxygen attached to the sulfonate moiety. The ratios of PSS to PEDOT were calculated using the integral area ratio of the peaks assigned to PEDOT and PSS. Specifically, the ratio of the S2p_3/2_ peak area of PSS relative to that of PEDOT can be used to estimate the relative composition of PSS to PEDOT at the film surface. The PSS to PEDOT surface composition ratio of the PEDOT:PSS and Te-PEDOT:PSS films was altered by H_2_SO_4_ treatment. The highest PSS/PEDOT surface composition ratios of 2.23 and 1.45 were found for the PEDOT:PSS and Te-PEDOT:PSS films without H_2_SO_4_ treatment, whereas increasing the H_2_SO_4_ concentration to 100 vol% reduced the PSS/PEDOT ratio to 1.28 and 1.34 for the treated PEDOT:PSS and Te-PEDOT:PSS films, respectively. H_2_SO_4_ treatment selectively removes the PSS component from the PEDOT:PSS and Te-PEDOT:PSS composites, leading to 43 and 8% reductions of the PSS content of the respective composites. The decreased PSS/PEDOT ratio is reflective of the structural rearrangement induced by the decrease in the PSS content of the composites. The change in the PSS content also influences the crystallinity of PEDOT:PSS and the concentration of charge carriers.

**Figure 3:**
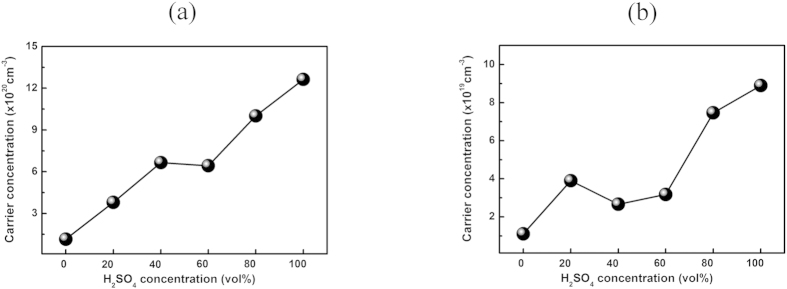
Carrier concentration of (**a**) PEDOT:PSS and (**b**) Te-PEDOT:PSS depending on the concentration of the H_2_SO_4_ solution used for treatment.

To evaluate the change in the charge carrier concentration of PEDOT:PSS and Te-PEDOT:PSS induced by H_2_SO_4_ treatment, Hall effect measurements were conducted. Figure 3 shows the change in the carrier concentration of the PEDOT:PSS and Te-PEDOT:PSS films in response to the H_2_SO_4_ concentration. The carrier concentration increased with increasing H_2_SO_4_ concentration for both PEDOT:PSS and Te-PEDOT:PSS. Interestingly, the carrier concentration increased abruptly after treatment with 80 and 100 vol% H_2_SO_4_^24^. The overall concentration of carriers in PEDOT:PSS was higher than that of Te-PEDOT:PSS because of the lower PEDOT:PSS content in Te-PEDOT:PSS. This increase in the carrier concentration due to H_2_SO_4_ treatment results from the change in the structure of PEDOT:PSS as a result of the decrease in the PSS content as explained in relation to the XPS results; this effect also increases the electrical conductivity. This result is also of great significance for manipulating the thermoelectric properties of PEDOT:PSS and Te-PEDOT:PSS, as described below.

### Seebeck coefficient

The Seebeck coefficient is a parameter that describes the fundamental electronic transport property of materials, and depends on the entropy of a carrier with unit charge. Specifically, it is a measure of the contributions of carriers to the conductivity at energy levels away from the Fermi level (*E*_F_). According to the Mott relationship, *S* is defined as:


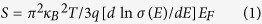


where *κ*_B_ is the Boltzmann constant and *E* is the electronic energy. In the framework of energy band theory and the Boltzmann equation, Equation (1) is transformed into:


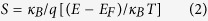


The increased carrier concentration will push *E*_F_ into the conduction band. This in turn leads to a reduced *S*^7^.

The PEDOT:PSS and Te-PEDOT:PSS films both exhibited a decrease in *S* (from 19.4 to 10.35 and 250 to 85.66 μV K^−1^, respectively) after treatment with 100 vol% H_2_SO_4_. The Hall effect measurement demonstrated that the carrier concentration increased with increasing H_2_SO_4_ concentration, as shown in Fig. 3. The increase in the carrier concentration due to H_2_SO_4_ treatment of PEDOT:PSS and Te-PEDOT:PSS may account for the decrease in the *S* value. This result is in good accordance with theoretical explanation presented above.

**Figure 4:**
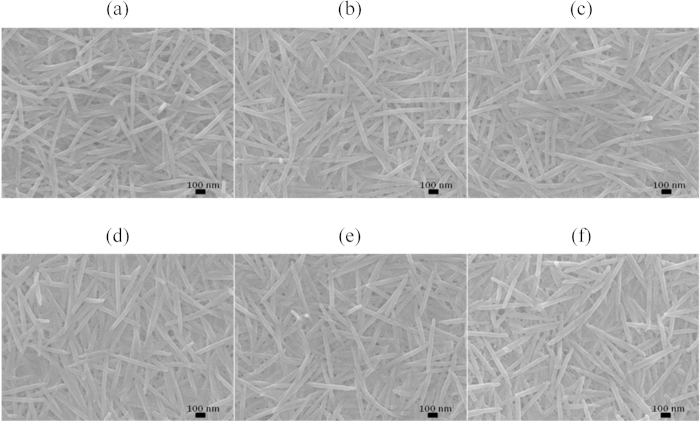
SEM images of surface of Te-PEDOT:PSS hybrid composite films treated with various concentrations of H_2_SO_4_. (**a**) 0, (**b**) 20, (**c**) 40, (**d**) 60, (**e**) 80, and (**f**) 100 vol%.

Figure 4 shows scanning electron microscope (SEM) images of the surface morphologies of the Te-PEDOT:PSS hybrid composite films after treatment with various concentrations of H_2_SO_4_. The surface of the Te-PEDOT:PSS film before H_2_SO_4_ treatment consists of composite nanorods that are densely packed together and interconnected with each other. The surface of the composite nanorods was also smooth and clear. It was confirmed that the surface structure of the Te composite nanorods was maintained even after treatment with 100 vol% H_2_SO_4_. Furthermore, the nanorods remained interconnected without the occurrence of chopping phenomena due to H_2_SO_4_ treatment. Therefore, the electrical conductivity of the Te-hybrid, which is mainly derived from the PEDOT:PSS component, could still increase with H_2_SO_4_ treatment given that the increase in the electrical conductivity arises from nanostructural rearrangement of PEDOT:PSS which is interconnected on the surface of the Te nanorods. However, as discussed, an increase in the overall carrier concentration of the Te composite nanorods can adversely influence the Seebeck effect. Atomic force microscope (AFM) images of PEDOT:PSS and Te-PEDOT:PSS (Figs S3 and S4) showed minimal morphological and structural changes due to acid treatment. We believe that the morphological stability of the Te structure even after H_2_SO_4_ treatment may be a significant factor contributing to the overall enhancement of the thermoelectric properties of Te-PEDOT:PSS.

### Flexible thermoelectric generator

**Figure 5:**
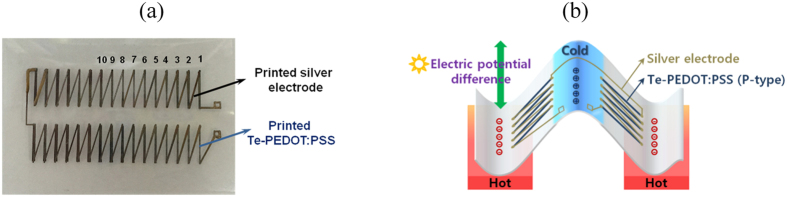
(**a**) Image of the planar thermoelectric generator consisting of 32 legs arranged in two rows and (**b**) schematic diagram of geometry of the invented thermoelectric generator and the generation of electricity.

**Figure 6:**
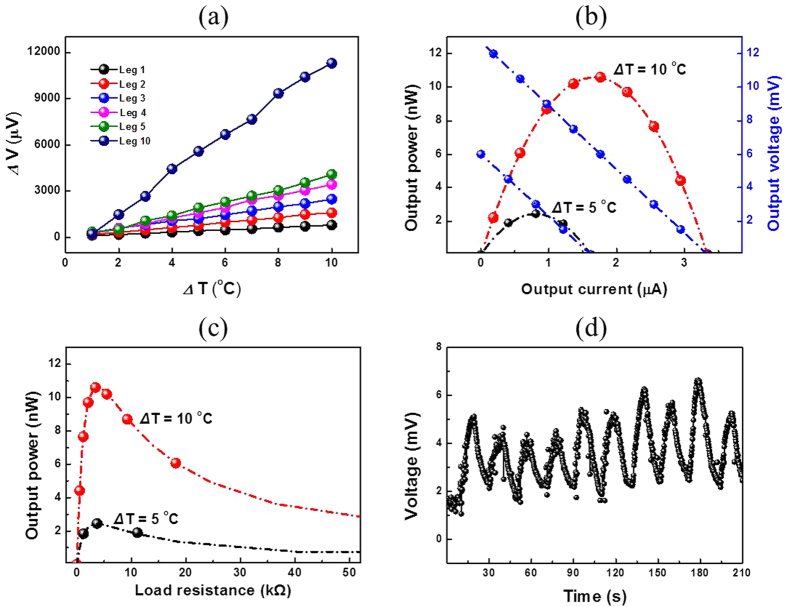
(**a**) Open circuit thermoelectric voltage (V_oc_) *vs.* temperature difference (Δ*T*) according to the number of TE legs, (**b**) output power curves depending on the output voltage and output current at Δ*T* = 5 and 10 °C, (**c**) output power curves according to the different load resistance at Δ*T* = 5 and 10 °C, and (**d**) the variation of output thermoelectric voltage with the cyclic change in temperature difference.

A solution of Te-PEDOT:PSS was used to fabricate flexible thermoelectric generators via a printing process. In order to maximize the thermoelectric efficiency, a planar type thermoelectric array with high density and high aspect ratio was designed (Fig. 5(a)). A thermoelectric generator comprising 32 legs arranged in two rows was elaborately printed by using the Te-PEDOT:PSS solution on a flexible substrate. The difference in the electric potential is derived from the temperature gradient between the edge and middle part of the thermoelectric array when the edge part makes contact with a heat source such as human skin, as shown in Fig. 5(b). The geometry of the thermoelectric generator invented in this study is easily adaptable to flexible and textile-embedded electronics to harness low waste-heat sources, such as human body heat. To evaluate the total thermoelectric output voltage of the integrated thermoelectric legs, the open circuit voltage (V_oc_) versus temperature difference (Δ*T*) based on the number of TE legs was measured as shown in Fig. 6(a). As the number of thermoelectric legs increased from 1 to 10, the output voltage of the printed thermoelectric generator increased proportionally (10-fold). Furthermore, the Seebeck voltage determined by the *V*_oc_ and Δ*T* of the printed thermoelectric generator is approximately 90 ± 10 *μ*V K^−1^, which corresponds to that of the acid treated Te-PEDOT:PSS thin film. Figure 6(b) shows the power output curves of the fabricated TEG depending on the output voltage and output current at Δ*T* = 5 and 10 °C. A row consisting of 16 legs of the fabricated Te-PEDOT:PSS TEG was used for measuring the power output. The fabricated Te-PEDOT:PSS TEG shows moderate output voltage of 12.75 mV and output power of 10.59 nW. As the temperature difference increased from 5 to 10 °C, the output voltage and output current proportionally increased twice, which led to increase in power output by four times. The internal resistance of Te-PEDOT:PSS TEG exhibited about 5 kΩ and a maximum power was observed when the internal resistance of Te-PEDOT:PSS was equal to the load resistance, as shown in Fig. 6(c).

In order to verify the practical use of the printed thermoelectric generator in thermal sensor application, a voltage response test was carried out. The voltage response depending on the temperature change was tracked by fixing both ends of the thermoelectric array onto an Al_2_O_3_ plate after slightly bending the thermoelectric array. The device on the plate was then placed on a hotplate with the temperature fixed to 30 ^o^C. An output thermoelectric voltage of 2 ± 0.2 mV was consistently generated from the 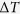 between the middle (air) and edge (hot plate) part of the thermoelectric generator. The built-up 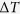 was estimated to be under 0.5 K from the Seebeck coefficient measured at room temperature. This small 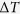 between the hot and cold part of the thermoelectric generator is possibly due to the low thermal conductivity of the polymer substrate and the underlying small temperature difference between the two parts. To further observe the sensitivity of the thermoelectric generator in response to a larger temperature difference, a glass rod was periodically brought into contact with the middle of the thermoelectric array at 10 s intervals to provide a colder environment in the middle part of the thermoelectric array. The output thermoelectric voltage repeatedly increased to 6 mV at 15 sec intervals in response to the manipulation of 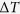 via contact with the glass rod, as shown in Fig. 6(d) and video 1 in the ESI. Based on this observation, it is confirmed that the fabricated thermoelectric generator operates properly and exhibits high sensitivity to temperature variation.

**Figure 7:**
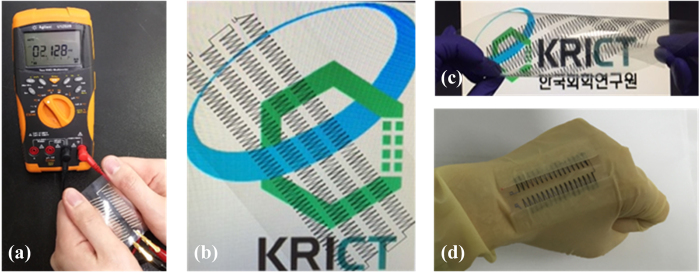
(**a**) Generation of electricity by grabbing both sides of the thermoelectric array at room temperature; (**b**,**c**) images of the flexible and twistable thermoelectric generator comprising 240 legs arranged in four rows, and (**d**) demonstration of the thermoelectric generator embedded in a glove for the generation of electricity by human body heat.

The electricity generated from the printed thermoelectric array was also measured by applying thermal energy from human hands in ambient atmosphere, as shown in Fig. 7(a) and video 2 in the ESI. The printed thermoelectric array generated a stable output voltage of over 2 mV when both sides of the array were grabbed, which is in good accordance with the results presented in Fig. 6(d). In addition, the fabricated thermoelectric generator could be easily bent and twisted. The design of the array makes this generator easy to embed in fabrics to effectively harness body heat as shown in Fig. 7(d), which is practically applicable to textile-based electronics.

## Conclusions

Te-PEDOT:PSS hybrid composites have a high Seebeck coefficient due to the nature of the Te nanrods; however, their electrical conductivity is unacceptably low. The thermoelectric properties of PEDOT:PSS and Te-PEDOT:PSS hybrid composites were successfully enhanced by simply immersing the thermoelectric materials into H_2_SO_4_ solutions of various concentrations, despite a slight decline in the Seebeck coefficient of the Te-PEDOT:PSS hybrid composite films. The enhanced electrical conductivity of H_2_SO_4_-treated PEDOT:PSS and Te-PEDOT:PSS is believed to arise from structural rearrangement of PEDOT:PSS due to the removal of PSS, which induces the formation of a more crystalline structure and increases the number of charge carriers. The power factor of H_2_SO_4_-treated PEDOT:PSS is much higher than that of non-treated PEDOT:PSS due to the notable enhancement of the electrical conductivity of H_2_SO_4_-treated PEDOT:PSS. Large area application was achieved using the organic species as demonstrated by the successful fabrication of flexible thermoelectric generators by a simple printing process using the treated Te-PEDOT:PSS composite having a high power factor of 284 μW m^−1^ K^−2^. The electrical power generation capability of the TEG was demonstrated with a maximum power output of 10.59 nW. The printed thermoelectric array generates a stable thermoelectric voltage of over 2 mV in response to human body heat and is expected to find utility in flexible thermoelectric devices with various device structures.

## Experimental

### Preparation of Te-PEDOT:PSS hybrid composite solutions

Sodium telluride (Na_2_TeO_3_, Aldrich) was used as a tellurium source. Firstly, 0.99 g ascorbic acid (Adrich) was dissolved in 40 mL of deionized water; 1 mL of PEDOT:PSS (Clevios PH 1000) solution filtered through a PVDF syringe filter (0.45 μm) was added to this solution. Subsequently, 0.07 g Na_2_TeO_3_ was added to the mixture under stirring. The temperature of the mixture was then increased to 90 °C and maintained for 20 h. Pure Te-PEDOT:PSS nanorods were collected by centrifugation of the reaction mixture at 9000 rpm for 10 min at least three times. The final product was resuspended in 5 mL of pure deionized water.

### Preparation of PEDOT:PSS and Te-PEDOT:PSS hybrid composite films

The PEDOT:PSS solutions were filtered by using a PVDF syringe filter to remove the large particles and the solution was then spin-coated on a glass substrate (70 mm × 50 mm) at 1000 rpm for 30 s and prebaked on a hot plate at 120 °C for 10 min to remove residual solvent. After prebaking, the PEDOT:PSS films were immersed in methanol (Samchun Pure Chemicals) for 10 min to optimize the initial electrical conductivity and were then annealed under the aforementioned conditions to finally form the 100 nm thick PEDOT:PSS film. The Te-PEDOT:PSS hybrid composite solutions were deposited on a glass substrate (30 mm × 10 mm) using a drop-casting method and slowly prebaked on a hot plate at 70 °C to generate films with a high-quality surface. After prebaking, the Te-PEDOT:PSS films were annealed at 120 °C to remove residual solvent and finally form the 1.1 μm thick Te-PEDOT:PSS film.

### Chemical treatment of PEDOT:PSS and Te-PEDOT:PSS hybrid composite films

For the chemical treatment, the PEDOT:PSS and Te-PEDOT:PSS films were immersed in H_2_SO_4_ (Samchun Pure Chemicals) solutions with various volume ratios (20–100%) for 10 min under ambient conditions. The films were then washed in methanol and annealed at 160 °C for 10 min.

### Fabrication of thermoelectric generators

Thirty two thermoelectric legs, which were arranged in two rows, were printed on a flexible PET substrate using a solution of the Te-PEDOT:PSS hybrid composite. For the printing process, the Te-PEDOT:PSS solution was dispensed through a 200 μm nozzle (SHOTMASTER 200 DS-S, Musashi Engineering, Inc., Japan). The printed thermoelectric arrays were prebaked on a hotplate at 120 ^o^C for 10 min and then treated with a concentration of 60 vol% H_2_SO_4_. For connection of the printed thermoelectric legs, metal electrodes were also dispensed using conductive silver paste (NB05, Soulbrain, Korea). The printed thermoelectric generator was then annealed at 150 ^o^C on a hotplate for 30 min to improve the electrical conductivity of the Ag electrodes and reduce the contact resistance between the Ag electrodes and thermoelectric elements. Each leg had dimensions of 1 mm × 1 cm and the overall size of the fabricated thermoelectric generator was 5 cm × 3 cm.

### Instrumentation

The electrical conductivity was measured via the four point probe method by using a combination of a Keithley 220 current source and a Keithley 195A digital multimeter. The Seebeck coefficient was measured by using the sample with silver electrodes with a home-built setup with a humidity of 18%. Two silver electrodes, 3 mm in width, were separated by a distance of 30 mm. The temperature gradient between the two electrodes was varied from 1 to 10 °C. All measurements were carried out a least five times for each sample as summarized in Table S1. The setup consisted of two Peltier devices to maintain controlled stages that could independently function as the hot part or cold part. For the measurement, a combination of a Keithley 2460 source meter, a Keithley 2700 multimeter, Keithley 6485 picoammeter/data acquisition system, a Keithley 2182A nanovoltameter, and a Keithley 2200-30-5 power supply was used. The nanocrystalline structure of the Te-PEDOT:PSS hybrid composite was evaluated via TEM (Tecnai G2, FEI, USA) analysis. The TEM samples were prepared by dropping the Te-PEDOT:PSS hybrid composite solution onto a copper grid. The surface morphology of the PEDOT:PSS and Te-PEDOT:PSS hybrid composite films was investigated using SEM (XL30S FEG, Philips, Netherlands) and AFM (Nanoscope IV, Digital Instruments, Korea) instruments over an area of 3 μm × 3 μm in non-contact mode. Before analysis of the films, platinum films with a thickness of approximately 100 nm were deposited on the prepared films with a coating machine (E-1030, Hitachi Ltd., Japan). The thickness of the films was determined by using an alpha-step surface profiler (α-step IQ, KLA Tencor, USA). The elements and compounds in the PEDOT:PSS and Te-PEDOT:PSS thin films were characterized by using XPS (AXIS NOVA, Kratos Analytical Ltd., UK). To compensate for the effects of surface charges, all binding energies were referenced to the C1s neutral carbon peak at 284.5 eV.

## Additional Information

**How to cite this article**: Jin Bae, E. *et al.* Enhancement of Thermoelectric Properties of PEDOT:PSS and Tellurium-PEDOT:PSS Hybrid Composites by Simple Chemical Treatment. *Sci. Rep.* **6**, 18805; doi: 10.1038/srep18805 (2016).

## Supplementary Material

Supplementary Information

Supplementary Video 1

Supplementary Video 2

## Figures and Tables

**Table 1:** Comparison between the room temperature (300 K) performance of as-reported Te nanostructure-polymer hybrid composite and the H_2_SO_4_-treated Te-PEDOT:PSS hybrid composite.

System	H_2_SO_4_ concentration (vol%)	Seebeck coefficient (μV K^−1^)	Electrical conductivity (S cm^−1^)	Power factor (μW m^−1^K^−2^)	Estimated *ZT*_max_	Reference
Te-PEDOT:PSS	—	163 (±4)	19.3 (±2.3)	70.9	0.1	23
Te-PEDOT:PSS	—	—	—	100		31
Te-PEDOT:PSS	—	~150	~0.2	~4.5		32
Chemically treated PEDOT:PSS	100	10.35	4839	51.85	0.08^a^	This work
	0	250	11.01	68.81		
	20	149.47	65.82	147.05		
Chemically treated Te-PEDOT:PSS	40	139.02	53.29	102.99		
	60	123.32	54.67	83.14		
	80	114.97	214.86	284	0.39^b^	
	100	85.66	334.68	245.58		

^a^Estimated from the thermal conductivity (0.20 W m^−1^K^−1^) of PEDOT:PSS^6^.

^b^Estimated from the thermal conductivity (0.22 W m^−1^K^−1^) of Te-PEDOT:PSS^23^.
