# Supplementary material for: Enhancement of Thermoelectric Properties of PEDOT:PSS and Tellurium-PEDOT:PSS Hybrid Composites by Simple Chemical Treatment
Source: Sci Rep. 2016 Jan 5;6:18805. doi: 10.1038/srep18805 (PMC4700464; doi:10.1038/srep18805)
Supplement: Supplementary Information [file srep18805-s1.doc]

**Supplementary Information**

**Enhancement of Thermoelectric Properties of PEDOT:PSS and Tellurium-PEDOT:PSS Hybrid Composites by Simple Chemical Treatment**

Eun Jin Bae,^†^ Young Hun Kang,^†^ Kwang-Suk Jang, and Song Yun Cho*****

*Division of Advanced Materials, Korea Research Institute of Chemical Technology, 141 Gajeong-ro, Yuseong-gu, Daejeon 34114, Republic of Korea*

**Additional Figures and Table**

(a)

(b)

(c)


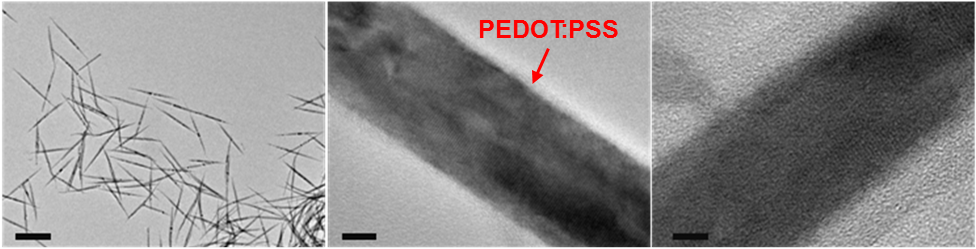


**Figure S1.** TEM images of Te-PEDOT:PSS hybrid composite: (a) 0.5 μm scale, (b) 10 nm scale, and (c) 5 nm scale.

(a)

(b)

**Figure S2.** *ΔV* *vs.* *ΔT* plot of (a) PEDOT:PSS treated with 100 vol% H_2_SO_4_ and (b) Te-PEDOT:PSS treated with 80 vol% H_2_SO_4_.


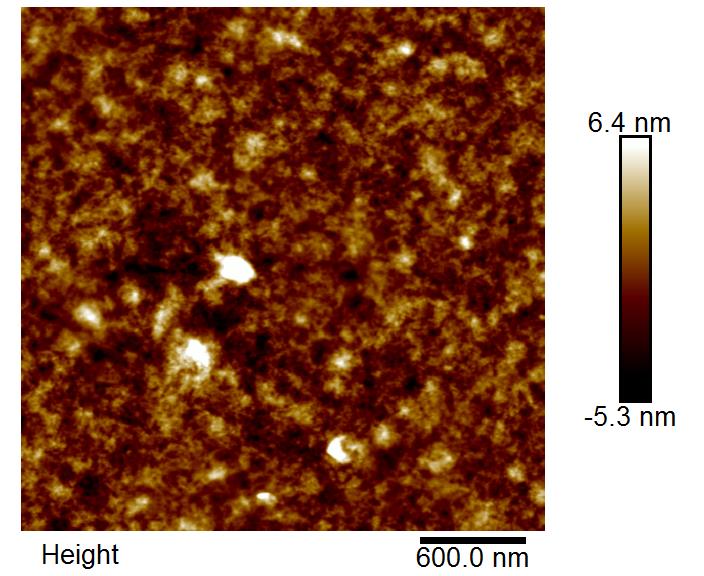

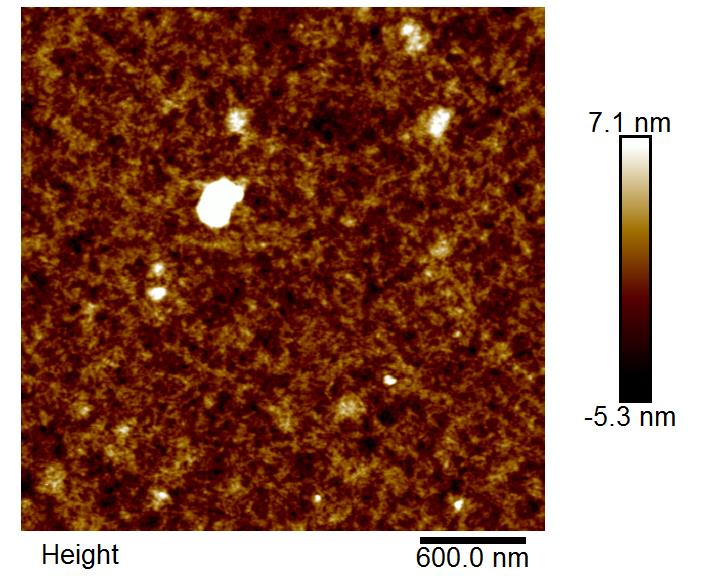

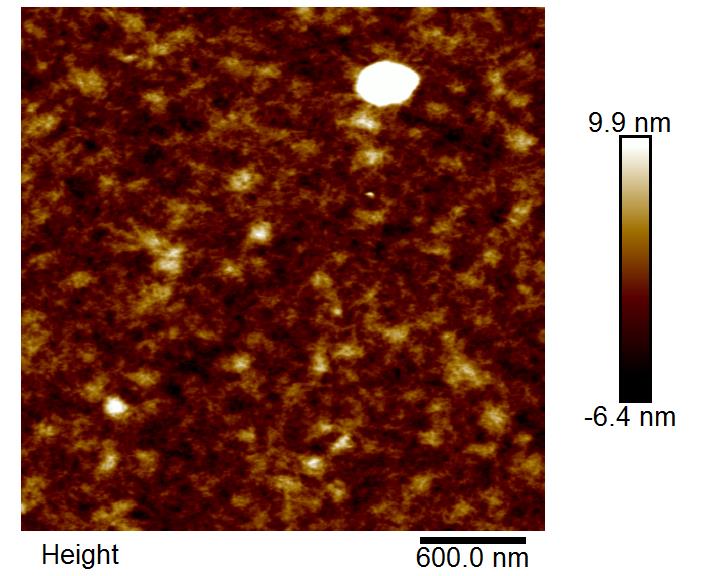


**600 nm**

**600 nm**

**600 nm**

(a)

(b)

(c)

(d)

(e)

(f)


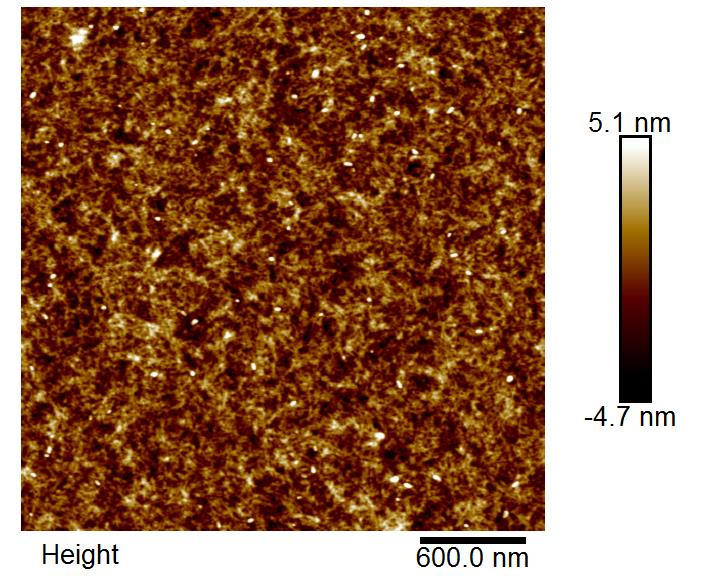

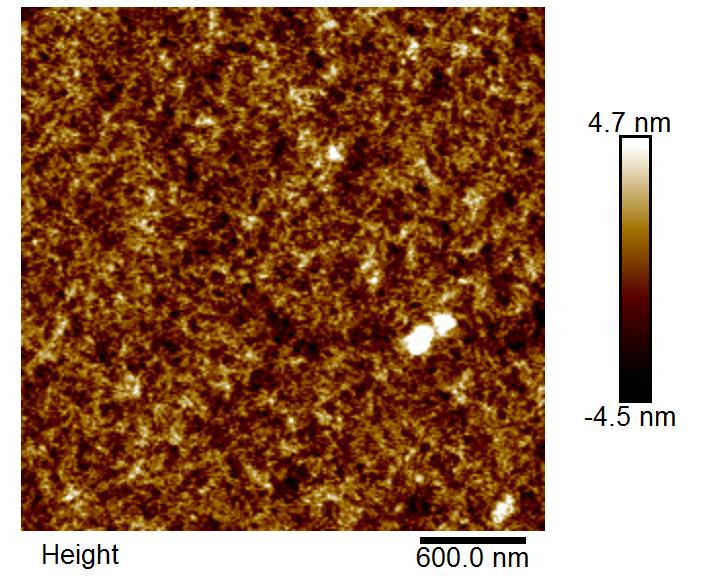

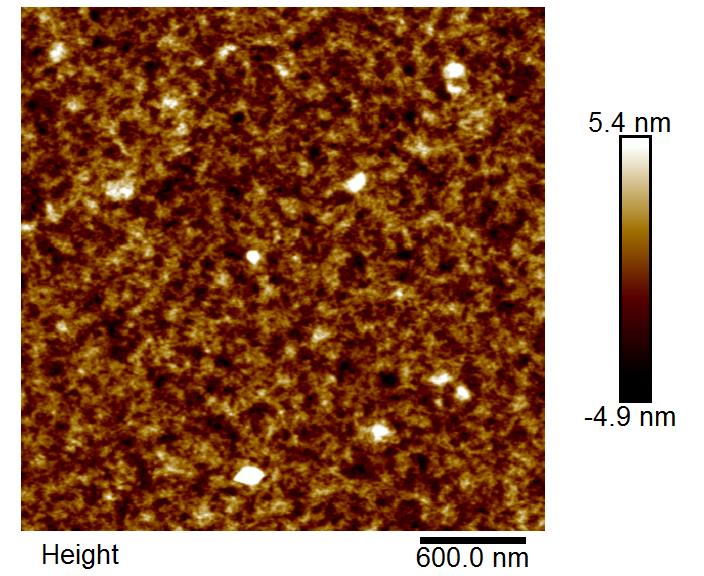


**600 nm**

**600 nm**

**600 nm**

**Figure S3.** AFM step profiler images of PEDOT:PSS thin films treated with various concentrations of H_2_SO_4_ : (a) 0, (b) 20, (c) 40, (d) 60, (e) 80, and (f) 100 vol%.


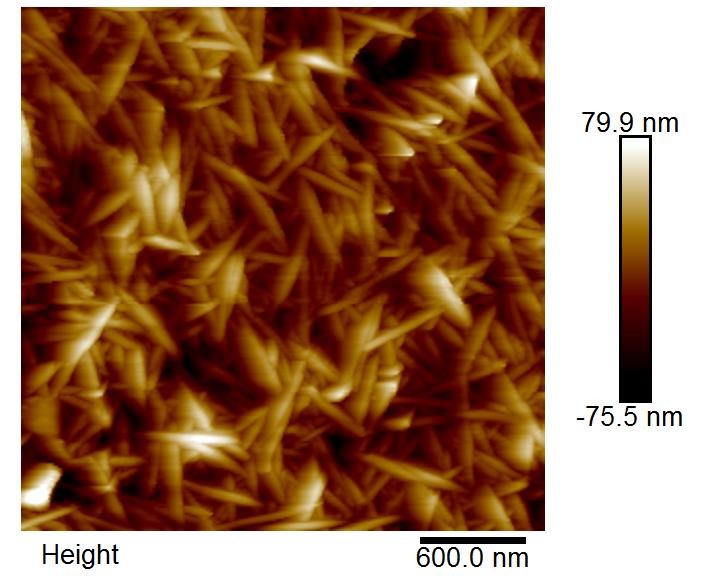

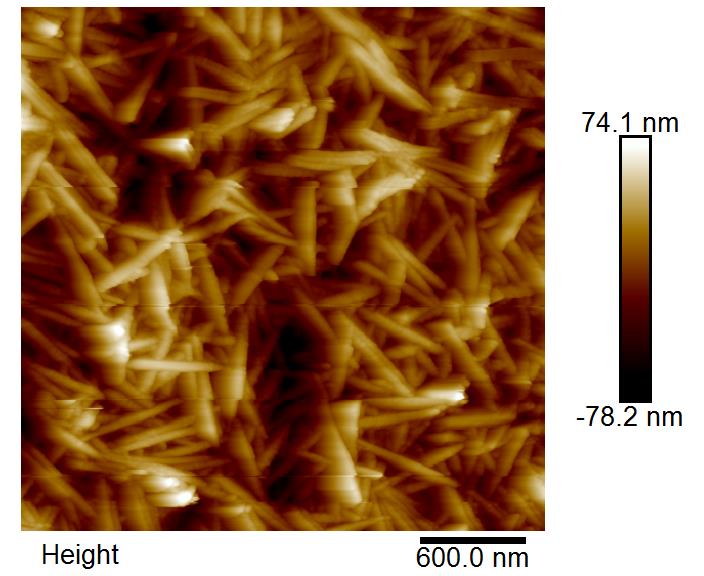

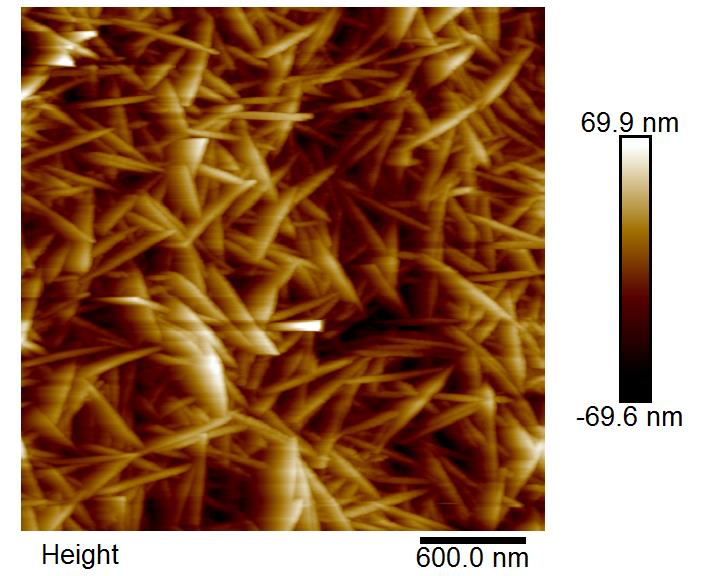


**600 nm**

**600 nm**

**600 nm**

(a)

(b)

(c)

(d)

(e)

(f)


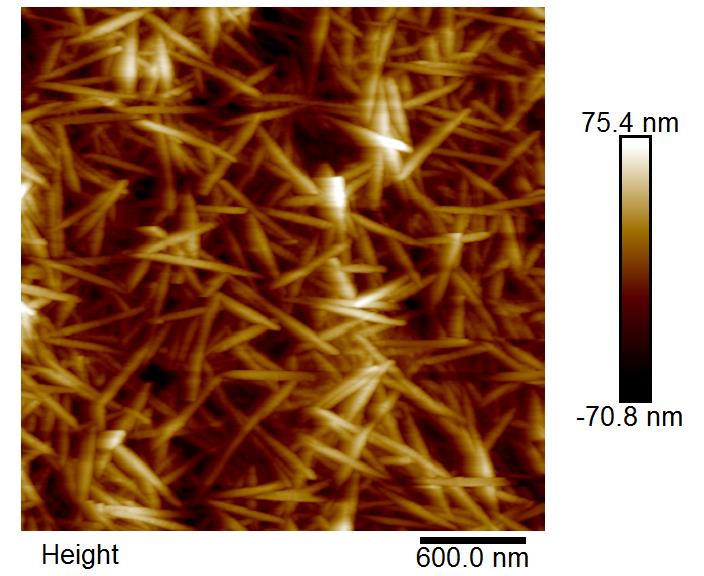

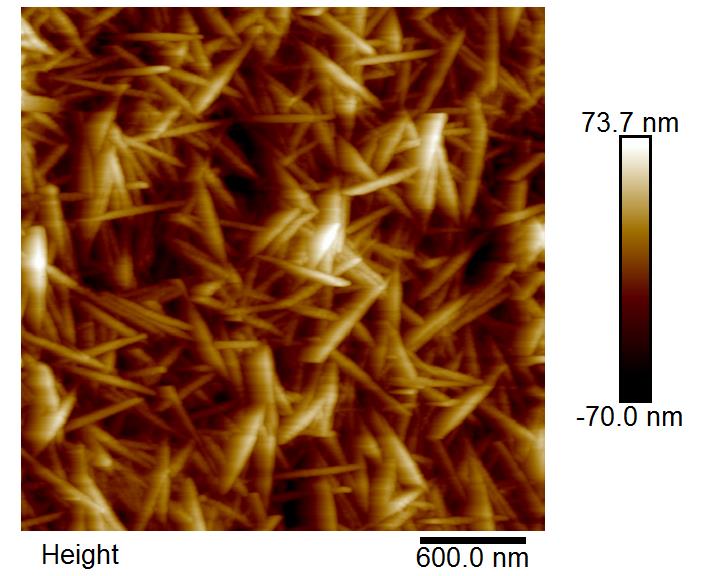

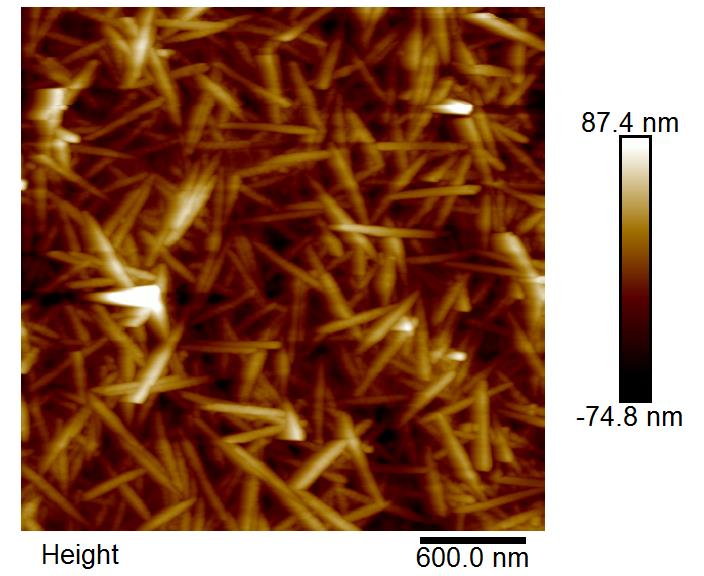


**600 nm**

**600 nm**

**600 nm**

**Figure S4.** AFM step profiler images of Te-PEDOT:PSS hybrid composite films treated with various concentrations of H_2_SO_4_: (a) 0, (b) 20, (c) 40, (d) 60, (e) 80, and (f) 100 vol%.

Table S1. Thermoelectric and electrical properties of the Te-PEDOT:PSS thin films depending on the synthetic batch

| H_2_SO_4_ treatment | Batch  number | Seebeck  coefficient  (μV K^-1^) | Electrical  conductivity  (S cm^-1^) | Power  factor  (μW m^-1^ K^-2^) |
| --- | --- | --- | --- | --- |
| without | 1 | 227.4±12.3 | 14.2±1.2 | 74.0±14.2 |
|  | 2 | 250.1±22.5 | 11.0±3.2 | 71.6±32.6 |
|  | 3 | 213.5±32.2 | 13.8±2.7 | 66.3±31.7 |
|  | 4 | 221.7±18.5 | 13.5±4.1 | 68.3±30.4 |
|  | 5 | 230.1±16.5 | 14.4±1.6 | 77.3±19.5 |
| with  80 vol% H_2_SO_4_ | 1 | 106.2±6.6 | 220.1±20.6 | 250.8±54.2 |
|  | 2 | 110.6±5.9 | 219.2±31.0 | 271.3±66.7 |
|  | 3 | 121.2±4.2 | 201.4±26.1 | 296.03±55.4 |
|  | 4 | 115.0±7.6 | 214.8±10.1 | 286.1±51.0 |
|  | 5 | 118.9±3.8 | 206.6±24.7 | 293.8±53.6 |
